# Association of SARS-CoV-2 nucleocapsid viral antigen and the receptor for advanced glycation end products with development of severe disease in patients presenting to the emergency department with COVID-19

**DOI:** 10.3389/fimmu.2023.1130821

**Published:** 2023-03-21

**Authors:** Zachary A. Matthay, Alexander T. Fields, Katherine D. Wick, Chayse Jones, H. Clifford Lane, Kimberly Herrera, Brenda Nuñez-Garcia, Efstathios Gennatas, Carolyn M. Hendrickson, Aaron E. Kornblith, Michael A. Matthay, Lucy Z. Kornblith, the COVID-19 Associated Coagulopathy Inflammation Thrombosis (Co-ACIT) Study Group, Biniam Ambachew

**Affiliations:** ^1^ Department of Surgery, Zuckerberg San Francisco General Hospital, University of California San Francisco, San Francisco, CA, United States; ^2^ Division of Pulmonary, Critical Care, Allergy and Sleep Medicine, Department of Medicine, University of California, San Francisco, San Francisco, CA, United States; ^3^ Cardiovascular Research Institute, University of California, San Francisco, San Francisco, CA, United States; ^4^ Division of Clinical Research, National Institute of Allergy and Infectious Diseases, National Institutes of Health, Bethesda, MD, United States; ^5^ Department of Epidemiology and Biostatistics, University of California San Francisco, San Francisco, CA, United States; ^6^ Department of Emergency Medicine, Zuckerberg San Francisco General Hospital, University of California San Francisco, San Francisco, CA, United States

**Keywords:** COVID-19, biomarkers, thromboinflammation, receptor for advanced glycation end products, triage

## Abstract

**Introduction:**

There remains a need to better identify patients at highest risk for developing severe Coronavirus Disease 2019 (COVID-19) as additional waves of the pandemic continue to impact hospital systems. We sought to characterize the association of receptor for advanced glycation end products (RAGE), SARS-CoV-2 nucleocapsid viral antigen, and a panel of thromboinflammatory biomarkers with development of severe disease in patients presenting to the emergency department with symptomatic COVID-19.

**Methods:**

Blood samples were collected on arrival from 77 patients with symptomatic COVID-19, and plasma levels of thromboinflammatory biomarkers were measured.

**Results:**

Differences in biomarkers between those who did and did not develop severe disease or death 7 days after presentation were analyzed. After adjustment for multiple comparisons, RAGE, SARS-CoV-2 nucleocapsid viral antigen, interleukin (IL)-6, IL-10 and tumor necrosis factor receptor (TNFR)-1 were significantly elevated in the group who developed severe disease (all *p*<0.05). In a multivariable regression model, RAGE and SARS-CoV-2 nucleocapsid viral antigen remained significant risk factors for development of severe disease (both *p*<0.05), and each had sensitivity and specificity >80% on cut-point analysis.

**Discussion:**

Elevated RAGE and SARS-CoV-2 nucleocapsid viral antigen on emergency department presentation are strongly associated with development of severe disease at 7 days. These findings are of clinical relevance for patient prognostication and triage as hospital systems continue to be overwhelmed. Further studies are warranted to determine the feasibility and utility of point-of care measurements of these biomarkers in the emergency department setting to improve patient prognostication and triage.

## 1 Introduction

The COVID-19 pandemic continues to present major risks to public health as additional waves and variants lead to new spikes in infections, at times overwhelming health care systems (1, 2). Although progress has been made in the prevention and treatment of COVID-19, a better understanding of early biologic markers predictive of ultimate disease severity may further facilitate patient prognostication and triage, and our understanding of the disease’s pathophysiology. Previous studies have identified a range of plasma biomarkers including those related to thromboinflammation, lung injury, and endothelial damage, that may be useful for predicting outcomes and understanding COVID-19 pathophysiology (3–7). However, most studies have focused on patients who have already been admitted to the hospital or intensive care unit, with a time lag between hospital presentation and sample collection. Data identifying biomarkers on patient presentation in the emergency department predictive of development of severe disease remains limited (8, 9). Common laboratory assays such as d-dimer, c-reactive protein, and fibrinogen may be of value, but may lack sensitivity and specificity to accurately predict COVID-19 outcomes (3, 10, 11).

The soluble receptor for advanced glycation end-products (RAGE) and SARS-CoV-2 nucleocapsid viral antigen are two biomarkers recently shown to have prognostic value in hospitalized patients with COVID-19 (12–14). However, few studies to date have examined these biomarkers in patients presenting to the emergency department. RAGE is an established biomarker of alveolar epithelial injury and is prognostic in patients with acute respiratory distress syndrome (ARDS) (15–17). Recent data from hospitalized patients with COVID-19 suggest that rises in biomarkers of alveolar epithelial injury including RAGE occur early in the disease course, whereas markers of endothelial injury manifest later (18). We therefore hypothesized that RAGE would be a useful early biomarker of disease severity in patients with COVID-19. Additionally, viral load measured by plasma SARS-CoV-2 ribonucleic acid (RNA) or by plasma SARS-CoV-2 nucleocapsid viral antigen is known to be associated with disease severity (19–22). However, to what extent plasma SARS-CoV-2 nucleocapsid viral antigen correlates with RAGE and with ultimate disease severity early in patients’ disease course is not well described. Further, understanding the association of SARS-CoV-2 nucleocapsid viral antigen plasma concentrations and associated thromboinflammatory biomarkers on arrival with patient outcome may inform clinical decision making in patients with COVID-19, and support future studies examining the role of point-of-care testing for these biomarkers.

The aims of this study were to test the prognostic value of RAGE and SARS-CoV-2 nucleocapsid viral antigen, as well as a panel of common thromboinflammatory biomarkers, measured on arrival to the emergency department in patients presenting with symptomatic COVID-19. We additionally aimed to examine the correlation between SARS-CoV-2 nucleocapsid antigen levels with a panel of thromboinflammatory biomarkers in this cohort.

## 2 Methods

### 2.1 Patient enrollment, data collection, inclusion/exclusion criteria

Participants included in this study were enrolled as part of the Covid-19 Associated Coagulopathy, Inflammation, and Thrombosis (Co-ACIT) Study (23). All adults presenting to our institution’s emergency department who were undergoing evaluation for COVID-19 were eligible. Study participants (n=125) were enrolled under an initial waiver of consent due to their respiratory compromise, and informed consent was obtained after initial blood collection. In addition to laboratory testing performed at the discretion of the treating clinician, whole blood was collected in sodium citrate (3.2%) on all patients for investigational assays. Exclusion criteria included patients who declined to participate (n=9), and those who were pregnant (n=1). After these exclusions, 115 patients remained in the study, and of those 77 tested positive for SARS-CoV-2 by reverse transcriptase PCR nasal swab testing and were included in this analysis (June 2020-March 2021). All patients were followed longitudinally to hospital discharge with comprehensive collection of clinical data including demographics, physiological and laboratory parameters, and clinical outcomes. The study was approved by the Committee on Human Research at the University of California, San Francisco (study number 20-30895).

### 2.2 Biomarker assays

Plasma samples were prepared by centrifugation from citrated whole blood collected on emergency department presentation and immediately stored at -80°C. RAGE, tissue plasminogen activator (tPA), plasminogen activator inhibitor 1 (PAI-1), soluble P-selectin, and protein C were measured by enzyme-linked immunosorbent assays (ELISA) using commercially available kits. Plasma SARS-CoV-2 nucleocapsid viral antigen levels were quantified using a single-molecule immune bead assay (Quanterix, Billerica, Massachusetts). D-dimer concentration, and percent activity of tissue plasminogen activator (tPA), plasminogen, antiplasmin, factor VIII, and von Willebrand Factor (vWF) were measured using STA Compact Max^®^ (Diagnostica Stago; Parsippany, New Jersey). Concentrations of interferon gamma inducible protein (IP-10), interleukin (IL)-10, vascular endothelial growth factor (VEGF), matrix metalloproteinase (MMP)-8, Thrombomodulin, surfactant protein D (SPD), angiopoietin (Ang)-1, Ang-2, and triggering receptor expressed on myeloid cells (TREM)-1 were measured using a multiplex magnetic bead immunoassay (Luminex, R&D Systems, Minneapolis, MN, USA). IL-6, IL-18, and tumor necrosis factor receptor (TNFR)-1 were measured using the Ella Immunoassay point of care system (Protein Simple-Biotechne, Minneapolis, Minnesota).

### 2.3 Statistical analysis

The primary outcome for this study was disease severity 7 days after presentation to the emergency department, classified by the World Health Organization’s (WHO) Ordinal Scale (24). This outcome was dichotomized as mild disease (WHO Scale 1-4; ambulatory/discharged or hospitalized but receiving no supplemental oxygen or only supplemental oxygen by mask/nasal cannula) versus severe disease or death (WHO scale 5-7; hospitalized and receiving non-invasive ventilation, high-flow nasal cannula, invasive mechanical ventilation, or death). Patient characteristics and biomarkers were compared between the mild and severe disease groups using Student’s *t* tests for normally distributed continuous variables, Wilcoxon-Rank Sum tests for non-normally distributed continuous variables, and Fisher’s exact test for binary variables. Biomarkers with non-normal distributions were log-transformed to reduce skew.

The association of biomarkers with development of severe disease at day 7 was assessed with bivariate analysis. After adjustment for multiple comparisons (Sidak-Holm adjusted P-Values (25)), the biomarkers showing significant association with development of severe disease or death were included in a multivariable model. The model additional included Acute Physiology and Chronic Health Evaluation III score (APACHE-III) to account physiologic and demographic predictors of poor outcome, and the final model was fit using backward stepwise logistic regression with significance set at 0.1 for retention. The APACHE-III score is a well validated tool to estimate mortality across a range of disease states including sepsis and acute respiratory distress syndrome (26, 27). Cut-point analysis using Youden’s index was used to determine optimal cutoffs for each of the statistically significant biomarkers for discriminating development of severe disease or death (28). The association of the SARS-CoV-2 nucleocapsid viral antigen levels with thromboinflammatory biomarkers was examined using spearman rank correlation coefficients.

## 3 Results

### 3.1 Patient characteristics

Of the 77 subjects included, 17 (22%) developed severe disease (n=15) or died (n=2) 7 days after presentation, while the remaining 60 (78%) of patients were ambulatory (n=43) or hospitalized with mild disease (n=17) (**Table 1**). Those who developed severe disease or death were older (mean age 70 vs 55 years, *p*<0.01), had higher rates of diabetes (59% vs 29%, *p*=0.04), but were not significantly different with respect to rates of kidney, liver, or heart disease (**Table 1**). Initial platelet counts, hemoglobin, international normalized ratio (INR), activated partial thromboplastic time, and creatinine were not different between the two groups, but fibrinogen and d-dimer were significantly elevated on presentation in those who developed severe disease or death (both *p*<0.05) (**Table 1**). APACHE-III scores on presentation were much higher in those who developed severe disease or death (59 vs 27 points, *p*<0.01) (**Table 1**). Eighteen percent (3/17) of patients who developed severe disease had venous thromboembolism (VTE) versus 3% (2/60) in those with mild disease (**Table 1**). Mortality at hospital discharge was 41% (7/17) in the patients who developed severe disease at 7 days, while one patient (2%) with mild disease at 7 days subsequently deteriorated and expired (*p*<0.01, **Table 1**).

**Table 1:** Characteristics of COVID-19 patients by ultimate disease severity.

	Ambulatory or hospitalized with mild disease	Severe disease or death	P-value
	N=60	N=17	
Age (years)	55 ( ± 17)	70 ( ± 12)	<0.01
Male Sex	60%	53%	0.78
Diabetes	29%	59%	0.04
Chronic Kidney Disease	6.9%	17.6%	0.19
Dialysis Dependence	3.4%	0.0%	0.99
Liver Failure or cirrhosis	5.0%	5.9%	0.99
Prior MI or CHF	8.3%	0.0%	0.58
Hemoglobin (g/dL)	14.2 (2.0)	14.3 (2.0)	0.87
Platelet Count (x10^9^/L)	236 (184–314)	203 (156-261)	0.09
International normalized ratio	1.0 (1.0-1.1)	1.1 (1.0-1.1)	0.17
PTT (s)	29 (25-31)	27 (25-31)	0.81
Fibrinogen, (mg/dL)	466 (347-579)	594 (507-720)	<0.01
D-dimer (ug/dL)	0.7 (0.4-1.4)	1.4 (1.0-2.5)	0.01
Creatinine (mg/dL)	0.8 (0.7-1.1)	0.8 (0.6-1.1)	0.81
Vent Free Days to 30 days	30.0 (30.0-30.0)	14.0 (3.0-30.0)	<0.01
Admission Acuity			<0.01
Emergency department only	45.0%	0.0%	
Floor	48.3%	11.8%	
Intensive care unit	6.7%	88.2%	
APACHE III Score	27 (17-42)	59 (52-73)	<0.01
Venous Thromboembolism	3%	18%	0.07
Disease Severity at Day 7 (WHO Ordinal Scale)			<0.01
Ambulatory	72%	0%	
Hospitalized, mild disease	28%	0%	
Hospitalized, severe disease	0%	88%	
Death	0%	12%	
Mortality at Discharge	1.7%	41.2%	<0.01

MI, myocardial infarction; CHF, congestive heart failure; APACHE-III, Acute Physiology and Chronic Health Evaluation III score; venous thromboembolism, deep vein thrombosis or pulmonary embolism.

### 3.2 Biomarkers compared by disease severity

There were several notable differences among thromboinflammatory biomarkers measured from the plasma collected at presentation between those who did and did not develop severe disease or death at day 7 (**Table 2**). The SARS-CoV-2 nucleocapsid viral antigen concentrations were notably higher among those who developed severe disease or death (median [IQR] 9.1 [8.5-9.8] log pg/ml vs 7.1 [5.3-8.2] log pg/ml, *p*<0.01) (**Table 2**). Eleven of the inflammatory and endothelial related biomarkers were also significantly elevated in those who developed severe disease or death, including RAGE, TREM-1, IL-6, IL-8, IL-10, IP-10, TNFR-1, MMP-8, and soluble P-selectin (all *p*<0.05) (**Table 2**). With respect to coagulation and fibrinolysis related markers, PAI-1 and vWF were significantly elevated in those who developed severe disease or death, but there were no differences in tPA, plasminogen, or antiplasmin (**Table 2**). After adjustment for multiple comparisons, SARS-CoV-2 nucleocapsid viral antigen, RAGE, IL-6, IL-10, and TNFR-1 retained statistical significance in association with development of severe disease or death (**Table 2**).

**Table 2:** Comparison of thromboinflammatory biomarkers among patients with COVID-19 by ultimate disease severity.

	Ambulatory or hospitalized with mild disease	Severe disease or death	Unadjusted p-value	Sidak-Holm Adjusted p-Value
Log SARS-CoV-2 Antigen (pg/ml)	7.1 (5.3-8.2)	9.1 (8.5-9.8)	<0.01	<0.01^*^
Inflammatory/Endothelial Biomarkers				
Log RAGE (pg/ml)	7.1 (6.5-7.9)	8.7 (8.3-9.1)	<0.01	<0.01^*^
Log TREM-1 (pg/ml)	5.2 (4.8-5.6)	5.7 (5.5-5.8)	<0.01	0.06
Log IL-6 (pg/ml)	3.2 (2.3-3.8)	4.4 (3.8-4.6)	<0.01	<0.01^*^
Log IL-8 (pg/ml)	2.5 (2.2-2.9)	3.0 (2.8-3.4)	<0.01	0.16
Log IL-10 (pg/ml)	2.2 (0.5-2.6)	2.9 (2.2-3.3)	<0.01	0.04^*^
Log IL-18 (pg/ml)	5.8 (5.4-6.2)	5.9 (5.6-6.1)	0.50	0.99
Log IP-10 (pg/ml)	5.9 (4.8-6.3)	6.1 (6.1-6.6)	0.01	0.18
Log TNFR1 (pg/ml)	7.1 (6.7-7.4)	7.6 (7.4-7.7)	<0.01	0.01^*^
Log MMP-8 (pg/ml)	7.5 (6.8-8.2)	8.6 (7.5-8.9)	0.02	0.24
Log Ang-1 (pg/ml)	8.5 (8.0-8.8)	8.7 (8.1-8.9)	0.90	0.99
Log Ang-2 (pg/ml)	7.1 (6.7-7.4)	7.2 (6.9-7.7)	0.32	0.97
Log P-selectin (ng/ml)	3.2 (2.8-3.6)	3.5 (3.4-3.9)	0.02	0.23
Log SPD (pg/ml)	8.3 (7.6-8.9)	8.4 (7.9-8.8)	0.62	0.99
Log VEGF (pg/ml)	3.1 (2.5-3.4)	3.1 (2.6-3.3)	0.82	0.99
Coagulation and Fibrinolysis Biomarkers				
Log Thrombomodulin (pg/ml)	8.4 (8.2-8.6)	8.7 (8.3-8.9)	0.16	0.85
Log PAI-1 (ng/ml)	1.4 (0.9-1.8)	1.8 (1.2-2.6)	0.02	0.21
Protein C (% normal)	89 (55-113)	60 (55-92)	0.11	0.78
von Willebrand Factor (% activity)	218 (119-340)	297 (212-420)	0.03	0.34
Factor VIII (% activity)	92 (60-208)	156 (89-280)	0.18	0.86
Log tPA (pg/ml)	7.7 (7.1-8.2)	7.9 (7.5-8.2)	0.52	0.99
Plasminogen (% activity)	126 (108-138)	114 (104-134)	0.58	0.99
Antiplasmin, (% activity)	110 (95-122)	108 (102-126)	0.69	0.99

*denotes statistical significance (p<0.05) after adjustment for multiple comparisons using Sidak-Holm method.

### 3.3 Multivariable model for severe/disease death

We then used stepwise modeling to build a multivariable model predictive of development of severe disease or death at 7 days, inputting biomarkers that remained significant after adjustment for multiple comparisons (SARS-CoV-2 nucleocapsid viral antigen, RAGE, IL-6, IL-10, and TNFR-1) as well as the APACHE-III score to adjust for clinical parameters of presenting disease severity. RAGE, SARS-CoV-2 nucleocapsid antigen, and APACHE-III score were retained in the final model as each being independent significant risk factors for development of severe disease or death at 7 days (**Table 3**). Cut-point analysis was performed for each of the five biomarkers that retained statistical significance after adjustment for multiple comparisons. This demonstrated that at cutoffs of >3,200pmg/ml for RAGE and 4,350pg/ml for SARS-CoV-2 viral antigen on emergency department presentation there was a sensitivity >80% and specificity >90% for development of severe disease or death at day 7 (**Table 4**). IL-6 and TNFR-1 each had good sensitivity (88% and 82% respectively), but lower specificity (72% and 70%) for stratifying patients by development of mild versus severe disease at day 7, and IL-10 was less discriminatory with sensitivity and specificity of 71% and 68% (**Table 4**).

**Table 3:** Multivariable model for risk factors for development of severe disease or death at 7 Days.

Variable	Odds Ratio	95% CI Low	95% CI high	P-Value
Log-RAGE^*^	1.38	1.02	1.85	0.03
Log-SARS-CoV-2 Viral Antigen^**^	3.26	1.07	9.99	0.04
APACHE-III Score^+^	2.90	1.28	6.57	0.01

*Receptor for advanced glycation end products, per 10 fold log (pg/ml).

**Per log (pg/ml), values below detection limit of the assay assigned value 0.1pg/ml.

+per 10 point change.

Pseudo R-squared = 0.75; n=71.

**Table 4:** Sensitivity and specificity of key biomarkers on presentation for predicting development of severe disease or death at 7 days using Youden index and cut-point analysis.

Biomarker	Cut-point*	Sensitivity	Specificity	AUC
Log RAGE	8.07 (3,197 pg/ml)	0.87	0.93	0.90
Log SARS-CoV-2 Viral Antigen	8.38 (4,359 pg/ml)	0.82	0.87	0.85
Log IL-6	3.66 (39 pg/ml)	0.88	0.72	0.80
Log TNFR-1	7.29 (1,466 pg/ml)	0.82	0.70	0.76
Log- IL-10	2.53 (13 pg/ml)	0.71	0.68	0.69

AUC,area under the receiver operator characteristic curve.

*Youden’s index to identify optimal cut-point, with natural log transformed raw values for each variable, corresponding raw concentration shown in parenthesis for reference.

### 3.4 Association of viral antigen and RAGE with thromboinflammatory biomarkers

**Figure 1:**
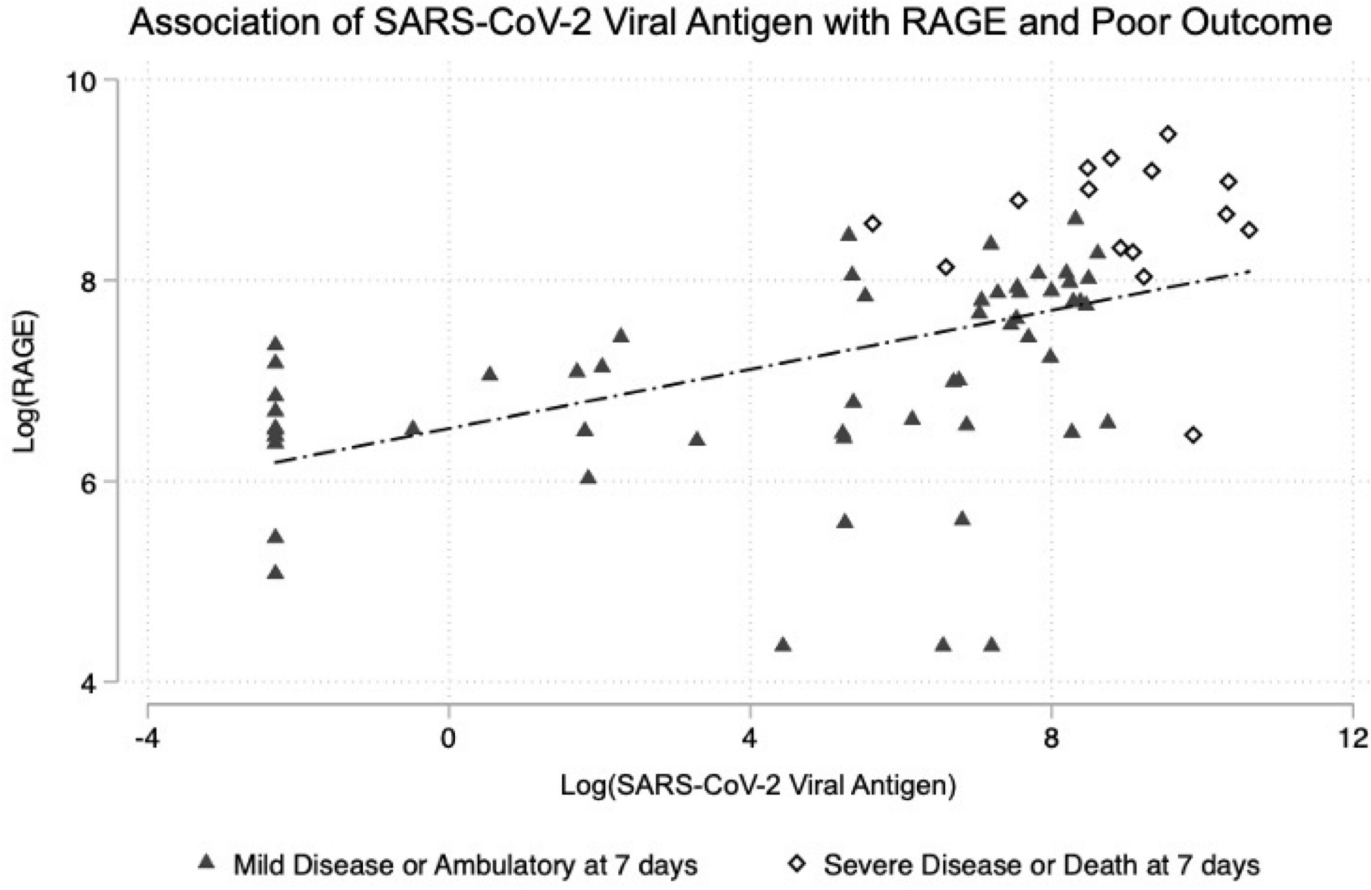
Association of SARS-CoV-2 viral antigen levels on emergency department presentation with RAGE and with subsequent clinical outcome at 7 days.

We then examined the association of the SARS-COV-2 nucleocapsid viral antigen and RAGE with each of the measured biomarkers. This reinforced the strong relationship between SARS-CoV-2 viral antigen with RAGE, and with poor outcome. Patients with severe disease or death clustered with both high RAGE and high viral antigen concentrations (**Figure 1**). IL-10, IP-10, and IL-6 were correlated significantly with both SARS-CoV-2 nucleocapsid viral antigen and RAGE (**Tables 5**, **6**). PAI-1 was significantly correlated with SARS-CoV-2 nucleocapsid viral antigen, but the trend was not statistically significant for RAGE (**Tables 5**, **6**). RAGE, but not SARS-CoV-2 nucleocapsid viral antigen, was also correlated with Von Willebrand Factor, TNFR-1, IL-18, and P-Selectin (**Table 6**).

**Table 5:** Spearman correlation coefficients of thromboinflammatory biomarkers with SARS-COV-2 nucleocapsid viral antigen.

Biomarker	Correlation Coefficient with Log-SAR-CoV-2 Viral Antigen	P-Value
Log IL-10 (pg/ml)	0.63	<0.01
Log IP-10 (pg/ml)	0.60	<0.01
Log RAGE (pg/ml)	0.60	<0.01
Log PAI-1 (ng/ml)	0.33	0.01
Log IL-6 (pg/ml)	0.30	0.02
Log SPD (pg/ml)	-0.24	0.06
Log TNFR-1 (pg/ml)	0.18	0.15
Log Thrombomodulin (pg/ml)	0.16	0.22
Plasminogen (% activity)	-0.16	0.23
Factor VIII (% activity)	-0.14	0.26
Log IL-8 (pg/ml)	0.14	0.29
Log IL-18 (pg/ml)	0.12	0.33
Von Willebrand Factory (% activity)	0.12	0.36
Log tPA (pg/ml)	-0.11	0.41
Log MMP-8 (pg/ml)	0.10	0.44
Antiplasmin (% activity)	0.07	0.57
Log TREM-1 (pg/ml)	0.05	0.67
Log Ang-2 (pg/ml)	-0.05	0.73
Log VEGF (pg/ml)	-0.04	0.75
Log P-Selectin (ng/ml)	0.00	0.98
Protein-C (% activity)	0.00	0.99
Log Ang-1 (pg/ml)	0.00	1.00

**Table 6:** Spearman correlation coefficients of thromboinflammatory biomarkers with RAGE.

Biomarker	Correlation Coefficient with Log-RAGE	P-Value
Log-SARS-CoV-2 Viral Antigen (pg/ml)	0.66	<0.01
Log IP-10 (pg/ml)	0.43	<0.01
Log IL-10 (pg/ml)	0.39	<0.01
Von Willebrand Factory (% activity)	0.37	<0.01
Log IL-6 (pg/ml)	0.34	<0.01
Log TNFR-1 (pg/ml)	0.29	0.01
Log IL-18 (pg/ml)	0.29	0.01
Log P-Selectin (ng/ml)	0.24	0.04
Log PAI-1 (ng/ml)	0.22	0.06
Log MMP-8 (pg/ml)	0.22	0.06
Antiplasmin (% activity)	0.22	0.07
Log IL-8 (pg/ml)	0.21	0.08
Factor VIII (% activity)	0.20	0.09
Protein-C (% activity)	-0.14	0.25
Log Ang-1 (pg/ml)	0.11	0.36
Log TREM-1 (pg/ml)	0.09	0.42
Log Ang-2 (pg/ml)	0.08	0.53
Log Thrombomodulin (pg/ml)	0.07	0.54
Log VEGF (pg/ml)	-0.06	0.62
Plasminogen (% activity)	0.05	0.69
Log SPD (pg/ml)	-0.03	0.78
Log tPA (pg/ml)	-0.01	0.97

## 4 Discussion

In this study, we examined a panel of biomarkers focusing on RAGE and SARS-CoV-2 nucleocapsid viral antigen in patients presenting to the emergency department with COVID-19. Our findings are consistent with prior literature demonstrating elevations in inflammatory cytokines and chemokines such as IL-6, IL-8, IL-10, IP-10, TNFR-1 and TREM-1 in the development of severe compared to mild COVID-19 (6, 8–10, 29–31). We also found increases in some but not all markers of thrombosis in those with severe disease including elevated PAI-1 and vWF activity. The strongest associations with development of severe disease and death were observed for RAGE, SARS-CoV-2 viral antigen, IL-6, IL-10, and TNFR-1. Our study adds to the existing literature because the measurements were performed on emergency department samples prior to any therapeutics and therefore the findings may be of prognostic value as these early measures of RAGE and SARS-CoV-2 viral antigen remained independent risk factors of development of severe disease or death in a multivariable model.

RAGE is an immunoglobulin prominently expressed by type I pneumocytes and is an established prognostic marker in patients with acute respiratory distress syndrome (ARDS) due to viral, bacterial and other causes (15–17, 32, 33). RAGE is also elevated in other states of lung injury such as emphysema, and in chronic diseases including diabetes and cardiovascular disease (32, 34). RAGE is thought to play a pathogenic role in ARDS based on animal studies (35, 36). Recent work identified that RAGE is also an important prognostic biomarker specifically in hospitalized patients with COVID-19 (3, 12, 13). However, few if any have reported on RAGE and ultimate disease severity when measured in patients early in their disease course. In our study, RAGE was the biomarker most strongly associated with development of severe disease or death at 7 days, suggesting this could be used as an important early prognostic biomarker in patients presenting with COVID-19 in the emergency department setting. Using the Youden index to determine an optimal cut-off value, a plasma RAGE concentration of greater than 3,200ng/ml had high sensitivity (87%) and specificity (93%) for later development of severe disease or death. This cutoff is similar to a threshold of 3,108ng/ml reported by Lim et al. (13) to stratify the need for mechanical ventilation, and lower than a threshold of 6,800ng/ml reported by Wick et al. for predicting mortality in hospitalized patients with COVID-19 (12).

A large cohort study examining changes in a broad range biomarkers in patients with COVID-19 found markers of alveolar epithelial injury rise early in the disease course while markers of endothelial injury occur later (18). This may explain why the observed differenced in markers of endothelial injury between those who developed mild versus severe disease were not as strong in this study, given that we only measured them at a single timepoint early in the disease course. This further underscores why markers of alveolar epithelial injury such as RAGE may be the best candidates for stratifying ultimate disease severity of COVID-19 at early timepoints. For example, no significant differences were observed for Ang-1, Ang-2 or VEGF in our study, although these are known to be increased in hospitalized patients with severe compared to mild COVID-19 (37). Further studies are needed examining how the relationship between the SARS-CoV-2 nucleocapsid viral antigen, RAGE, and additional biomarkers varies over time, which was not performed as part of this investigation.

TREM-1, a mediator of inflammation and prognostic biomarker in sepsis, was elevated in patients who developed severe disease, though did not achieve statistical significance after adjustment for multiple comparisons (38, 39). Recent data in emergency department patients demonstrated TREM-1 had excellent predictive ability for patients developing severe disease (defined as intubation or mortality within 30 days), with an AUC of 0.86 (8). This relationship was not as strong in our study, but the observed trend was the same, and a larger cohort may be needed to better examine these trends.

Our study also demonstrates a strong association of SARS-CoV-2 nucleocapsid viral antigen with development of severe disease in COVID-19, consistent with several prior studies of hospitalized patients (14, 19, 20, 40). Recent data from a large multicenter clinical trial consortium demonstrated that in patients hospitalized with COVID-19, higher SARS-CoV-2 nucleocapsid viral antigen measured within the first 72 hours was associated with worse clinical outcomes at 7 days (19). At a threshold of 1,000 pg/ml, the viral antigen was 77% sensitive and 59% specific for a worse outcome on the WHO ordinal scale at 7 days. Similarly, in an analysis of data from the Accelerating COVID-19 Therapeutic Interventions and Vaccines (ACTIV)-3 Therapeutics for Inpatients With COVID-19 (TICO) study group, a threshold of 1000ng/ml was associated with a 5 fold increased odds of worse respiratory status at 5 days and longer length of stay, but these levels were drawn 1-2 days after hospital admission (14). In our study, we identified a threshold of 4,500pg/ml to be very specific (82%) and sensitive (90%) for development of poor outcome at 7 days on the WHO ordinal scale. Although these findings should be validated in a larger cohort of patients, it is further notable that of the 11 patients who presented with a positive COVID-19 nasopharyngeal PCR test but had an undetectable viral SARS-COV-2 nucleocapsid viral antigen level, none developed severe disease or died at 7 days. A quantitative point of care test for the SARS-CoV-2 viral antigen was recently developed and shown to be accurate (41), broadening the potential clinical utility of the data presented in this study.

This study has several important limitations. First, our sample size is modest with 77 patients of whom 17 developed the primary outcome of severe disease or death at 7 days. Therefore, our findings of the potential prognostic value of RAGE and SARS-CoV-2 nucleocapsid viral antigen plasma levels need further validation in larger cohorts. This study was not a time to event analysis- the time to development of severe disease was not factored into our models, and a small proportion of patients (n=4) developed severe disease but recovered by day 7 thus were encompassed in the mild/ambulatory group. We also had limited statistical power to evaluate secondary outcomes such as venous thromboembolism due to the low absolute number of patients developing these outcomes. This analysis was exploratory and observational in nature- we tested a range of relevant thromboinflammatory biomarkers resulting in multiple hypothesis testing. To account for this, *p*-values were corrected using the conservative Sidak-Holm method, which is an appropriate but conservative method and may have resulted in type II errors (25). There was a higher proportion of patients with diabetes and older age in the severe disease group, which could be a source of bias in our results given RAGE may be elevated in chronic disease and advanced age (34, 42). However, we adjusted for this possible confounding in our multivariable model using the APACHE-III score which includes age as part of the input, and on additional sensitivity analyses no changes to our results were observed when controlling directly for age and diabetes in our multivariable model. Our study population included some but not all patients presenting to the emergency department with symptomatic COVID-19. Some of the sickest patients who may be rapidly triaged to the intensive care unit would not have been captured by our recruitment process. The focus of our study was to evaluate potential prognostic markers where clinical equipoise exists, and therefore omission of patients clearly critically-ill was desirable for our study’s purposes. Most patients in this study were enrolled prior to the widespread Omicron variant of SARS-CoV-2. Vaccination status of patients was also not included, but the majority of patients were enrolled prior to the implementation of the COVID-19 vaccine.

In conclusion, our study demonstrates the potential prognostic value of two emerging biomarkers predictive of development of severe disease in patients presenting to the emergency department for symptomatic COVID-19: RAGE and the SARS-COV-2 nucleocapsid viral antigen. These findings are highly clinically relevant given the observed relationship was identified on emergency department presentation early in patients’ disease course and remained significant even after controlling for physiologic measures of disease severity. Additional studies validating these findings and incorporating point of care assays for RAGE and SARS-COV-2 nucleocapsid viral antigen are warranted to further characterize their prognostic value and determine optimal discriminatory thresholds for patient risk stratification.

## Data availability statement

The raw data supporting the conclusions of this article will be made available by the authors, without undue reservation.

## Ethics statement

The studies involving human participants were reviewed and approved by Committee on Human Research at the University of California, San Francisco (study number 20-30895). The patients/participants provided their written informed consent to participate in this study.

## CO-ACIT study group members

Biniam Ambachew, Roland J. Bainton, Sarah Cary, Lauren Chalwell, Christopher Colwell, Clayton Josephy, Philip Kurien, Deanna Lee, Matthieu LeGrand, Juan Carlos Montoy, Viet Nguyen, John J. Park, Arun Prakash, Brittany Robinson, India Shelley.

## Author contributions

All authors contributed to the article and approved the submitted version. ZM performed study design, biomarker assays, analyzed data, and prepared the manuscript. AF assisted in study design, biomarker assays, analyzed data and provided revisions of the manuscript. KW performed biomarker assays, analyzed data, and provided revisions of the manuscript. CJ performed biomarker assays and analyzed data. KH and BN-G enrolled patients in the study, collected blood samples and performed research assays, and collected clinical data. CH, AK, MM assisted in study design and provided critical revisions of the manuscript. HL performed biomarker assays. LK performed study design, analyzed data, and helped prepared the manuscript. Members of the CO-ACIT study group enrolled patients in the study, collected blood samples and performed research assays, collected clinical data, and assisted with study design and execution.

## References

[1] Alexandridi M Mazej J Palermo E Hiscott J. The coronavirus pandemic – 2022: Viruses, variants & vaccines. Cytokine Growth Factor Rev (2022) 63:1–9. doi: 10.1016/j.cytogfr.2022.02.002 35216872 PMC8839804

[2] World Health Organization. Strategic preparedness, readiness and response plan to end the global COVID-19 emergency in 2022. World Health Organization (2022). Available at: https://scholar-google-com.ucsf.idm.oclc.org/scholar?q=related:1B3XMBv3QowJ:scholar.google.com/&scioq=covid+19+healthcare+supply+2022&hl=en&as_sdt=0,5#:~:text=All%204%20versions-,%5BPDF%5D%20who.int,-%5BPDF%5D%20COVID.

[3] Abers MS Delmonte OM Ricotta EE Fintzi J Fink DL de Jesus AAA. An immune-based biomarker signature is associated with mortality in COVID-19 patients. JCI Insight (2021) 6(1):144455. doi: 10.1172/jci.insight.144455 33232303 PMC7821609

[4] Leisman DE Mehta A Thompson BT Charland NC Gonye ALK Gushterova I. Alveolar, endothelial, and organ injury marker dynamics in severe COVID-19. Am J Respir Crit Care Med. (2022) 205(5):507–519. doi: 10.1164/rccm.202106-1514OC. PMC8906476 34878969

[5] Schneider M. The role of biomarkers in hospitalized COVID-19 patients with systemic manifestations. biomark Insights (2022) 17:11772719221108908. doi: 10.1177/11772719221108909 PMC9243490 35783222

[6] Ahmad R Haque M. Surviving the storm: Cytokine biosignature in SARS-CoV-2 severity prediction. Vaccines (Basel) (2022) 10(4):614. doi: 10.3390/vaccines10040614 35455363 PMC9026643

[7] Han H Ma Q Li C Liu R Zhao L Wang W. Profiling serum cytokines in COVID-19 patients reveals IL-6 and IL-10 are disease severity predictors. Emerg Microbes Infect (2020) 9(1):1123–30. doi: 10.1080/22221751.2020.1770129 PMC7473317 32475230

[8] Van Singer M Brahier T Ngai M Wright J Weckman AM Erice C. COVID-19 risk stratification algorithms based on sTREM-1 and IL-6 in emergency department. J Allergy Clin Immunol (2021) 147(1):99–106.e4. doi: 10.1016/j.jaci.2020.10.001 33045281 PMC7546666

[9] Goswami J MacArthur TA Sridharan M Tange J Kirmse AJ Lundell KA. Biomarkers of thromboinflammation correlate to COVID-19 infection and admission status in emergency department patients. Thromb Update (2021) 5:100090. doi: 10.1016/j.tru.2021.100090 PMC8603399 38620680

[10] Ceci FM Fiore M Gavaruzzi F Angeloni A Lucarelli M Scagnolari C. Early routine biomarkers of SARS-CoV-2 morbidity and mortality: Outcomes from an emergency section. Diagnostics. (2022) 12(1):176. doi: 10.3390/diagnostics12010176 35054342 PMC8774587

[11] Hariyanto TI Japar KV Kwenandar F Damay V Siregar JI Lugito NPH. Inflammatory and hematologic markers as predictors of severe outcomes in COVID-19 infection: A systematic review and meta-analysis. Am J Emergency Med (2021) 41:110–9. doi: 10.1016/j.ajem.2020.12.076 PMC7831442 33418211

[12] Wick KD Siegel L Neaton JD Oldmixon C Lundgren J Dewar RL. RAGE has potential pathogenetic and prognostic value in nonintubated hospitalized patients with COVID-19. JCI Insight (2022) 7(9):e157499. doi: 10.1172/jci.insight.157499 35298440 PMC9090244

[13] Lim A Radujkovic A Weigand MA Merle U. Soluble receptor for advanced glycation end products (sRAGE) as a biomarker of COVID-19 disease severity and indicator of the need for mechanical ventilation, ARDS and mortality. Ann Intensive Care (2021) 11(1):50. doi: 10.1186/s13613-021-00836-2 33751264 PMC7983090

[14] ACTIV-3/TICO Study Group. The association of baseline plasma SARS-CoV-2 nucleocapsid antigen level and outcomes in patients hospitalized with COVID-19. Ann Intern Med (2022) 175(10):1401–1410. doi: 10.7326/M22-0924 PMC9447373 36037469

[15] Shirasawa M Fujiwara N Hirabayashi S Ohno H Iida J Makita K. Receptor for advanced glycation end-products is a marker of type I lung alveolar cells. Genes to Cells (2004) 9(2):165–74. doi: 10.1111/j.1356-9597.2004.00712.x 15009093

[16] Uchida T Shirasawa M Ware LB Kojima K Hata Y Makita K. Receptor for advanced glycation end-products is a marker of type I cell injury in acute lung injury. Am J Respir Crit Care Med (2006) 173(9):1008–15. doi: 10.1164/rccm.200509-1477OC PMC2662912 16456142

[17] Jabaudon M Blondonnet R Pereira B Cartin-Ceba R Lichtenstern C Mauri T. Plasma sRAGE is independently associated with increased mortality in ARDS: a meta-analysis of individual patient data. Intensive Care Med. (2018) 44(9):1388–1399. doi: 10.1007/s00134-018-5327-1 PMC6132684 30051136

[18] Leisman DE Mehta A Thompson BT Charland NC Gonye ALK Gushterova I. Alveolar, endothelial, and organ injury marker dynamics in severe COVID-19. Am J Respir Crit Care Med (2022) 205(5):507–19. doi: 10.1164/rccm.202106-1514OC PMC8906476 34878969

[19] Wick KD Leligdowicz A Willmore A Carrillo SA Ghale R Jauregui A. Plasma SARS-CoV-2 nucleocapsid antigen levels are associated with progression to severe disease in hospitalized COVID-19. Crit Care (2022) 26(1):278. doi: 10.1186/s13054-022-04153-3 36104754 PMC9472195

[20] Brasen CL Christensen H Olsen DA Kahns S Andersen RF Madsen JB. Daily monitoring of viral load measured as SARS-CoV-2 antigen and RNA in blood, IL-6, CRP and complement C3d predicts outcome in patients hospitalized with COVID-19. Clin Chem Lab Med (2021) 59(12):1988–97. doi: 10.1515/cclm-2021-0694 34455731

[21] Fajnzylber J Regan J Coxen K Corry H Wong C Rosenthal A. SARS-CoV-2 viral load is associated with increased disease severity and mortality. Nat Commun (2020) 11(1):5493. doi: 10.1038/s41467-020-19057-5 33127906 PMC7603483

[22] Salto-Alejandre S Berastegui-Cabrera J Camacho-Martínez P Infante-Domínguez C Carretero-Ledesma M Crespo-Rivas JC. SARS-CoV-2 viral load in nasopharyngeal swabs is not an independent predictor of unfavorable outcome. Sci Rep. (2021) 11(1):12931. doi: 10.1038/s41598-021-92400-y 34155307 PMC8217169

[23] Joffre J Rodriguez L Matthay ZA Lloyd E Fields AT Bainton RJ. COVID-19-Associated lung microvascular endotheliopathy: A “From the bench” perspective. Am J Respir Crit Care Med (2022) 206(8):961–972. doi: 10.1164/rccm.202107-1774OC PMC9801996 35649173

[24] Covid-19-therapeutic-trial-synopsis.pdf . Available at: https://cdn.who.int/media/docs/default-source/blue-print/covid-19-therapeutic-trial-synopsis.pdf?sfvrsn=44b83344_1&download=true.

[25] Aickin M Gensler H. Adjusting for multiple testing when reporting research results: the bonferroni vs Holm methods. Am J Public Health (1996) 86(5):726–8. doi: 10.2105/AJPH.86.5.726 PMC1380484 8629727

[26] Czajka S Ziębińska K Marczenko K Posmyk B Szczepańska AJ Krzych ŁJ. Validation of APACHE II, APACHE III and SAPS II scores in in-hospital and one year mortality prediction in a mixed intensive care unit in Poland: a cohort study. BMC Anesthesiol (2020) 20(1):296. doi: 10.1186/s12871-020-01203-7 33267777 PMC7709291

[27] Knaus WA Wagner DP Draper EA Zimmerman JE Bergner M Bastos PG. The APACHE III prognostic system. risk prediction of hospital mortality for critically ill hospitalized adults. Chest. (1991) 100(6):1619–36. doi: 10.1378/chest.100.6.1619 1959406

[28] Fluss R Faraggi D Reiser B. Estimation of the youden index and its associated cutoff point. Biom J (2005) 47(4):458–72. doi: 10.1002/bimj.200410135 16161804

[29] Herold T Jurinovic V Arnreich C Lipworth BJ Hellmuth JC von Bergwelt-Baildon M. Elevated levels of IL-6 and CRP predict the need for mechanical ventilation in COVID-19. J Allergy Clin Immunol (2020) 146(1):128-136.e4. doi: 10.1016/j.jaci.2020.05.008 32425269 PMC7233239

[30] Kerget F Kerget B İba Yılmaz S Kızıltunç A. Evaluation of the relationship between TREM-1/TREM-2 ratio and clinical course in COVID-19 pneumonia. Int J Clin Pract (2021) 75(10):e14697. doi: 10.1111/ijcp.14697 34365706 PMC8420347

[31] Zhang Z Ai G Chen L Liu S Gong C Zhu X. Associations of immunological features with COVID-19 severity: a systematic review and meta-analysis. BMC Infect Dis (2021) 21(1):738. doi: 10.1186/s12879-021-06457-1 34344353 PMC8329624

[32] Chiappalupi S Salvadori L Vukasinovic A Donato R Sorci G Riuzzi F. Targeting RAGE to prevent SARS-CoV-2-mediated multiple organ failure: Hypotheses and perspectives. Life Sci (2021) 272:119251. doi: 10.1016/j.lfs.2021.119251 33636175 PMC7900755

[33] Narvaez-Rivera RM Rendon A Salinas-Carmona MC Rosas-Taraco AG. Soluble RAGE as a severity marker in community acquired pneumonia associated sepsis. BMC Infect Diseases (2012) 12(1):15. doi: 10.1186/1471-2334-12-15 22264245 PMC3274434

[34] Sabbatinelli J Castiglione S Macrì F Giuliani A Ramini D Vinci MC. Circulating levels of AGEs and soluble RAGE isoforms are associated with all-cause mortality and development of cardiovascular complications in type 2 diabetes: a retrospective cohort study. Cardiovasc Diabetol (2022) 21:95. doi: 10.1186/s12933-022-01535-3 35668468 PMC9169316

[35] Blondonnet R Audard J Belville C Clairefond G Lutz J Bouvier D. RAGE inhibition reduces acute lung injury in mice. Sci Rep (2017) 7(1):7208. doi: 10.1038/s41598-017-07638-2 28775380 PMC5543147

[36] Jabaudon M Blondonnet R Roszyk L Bouvier D Audard J Clairefond G. Soluble receptor for advanced glycation end-products predicts impaired alveolar fluid clearance in acute respiratory distress syndrome. Am J Respir Crit Care Med (2015) 192(2):191–9. doi: 10.1164/rccm.201501-0020OC 25932660

[37] Relationship between COVID-19 severity, markers of endothelial impairment, and simple covid risk index . Available at: https://www.mp.pl/paim/issue/article/16348. 10.20452/pamw.16348 36169165

[38] Bouchon A Dietrich J Colonna M. Cutting edge: Inflammatory responses can be triggered by TREM-1, a novel receptor expressed on neutrophils and Monocytes1. J Immunol (2000) 164(10):4991–5. doi: 10.4049/jimmunol.164.10.4991 10799849

[39] Brenner T Uhle F Fleming T Wieland M Schmoch T Schmitt F. Soluble TREM-1 as a diagnostic and prognostic biomarker in patients with septic shock: an observational clinical study. Biomarkers. (2017) 22(1):63–9. doi: 10.1080/1354750X.2016.1204005 27319606

[40] Wang H Hogan CA Verghese M Solis D Sibai M Huang C. SARS-CoV-2 nucleocapsid plasma antigen for diagnosis and monitoring of COVID-19. Clin Chem (2021) 68(1):204–13. doi: 10.1093/clinchem/hvab216 PMC8522398 34605900

[41] Heggestad JT Kinnamon DS Olson LB Liu J Kelly G Wall SA. Multiplexed, quantitative serological profiling of COVID-19 from blood by a point-of-care test. Sci Adv (2021) 7(26):eabg4901. doi: 10.1126/sciadv.abg4901 34172447 PMC8232907

[42] Steenbeke M De Bruyne S De Buyzere M Lapauw B Speeckaert R Petrovic M. The role of soluble receptor for advanced glycation end-products (sRAGE) in the general population and patients with diabetes mellitus with a focus on renal function and overall outcome. Crit Rev Clin Lab Sci (2021) 58(2):113–30. doi: 10.1080/10408363.2020.1791045 32669010

